# Robust multi-label surgical tool classification in noisy endoscopic videos

**DOI:** 10.1038/s41598-024-82351-5

**Published:** 2025-02-14

**Authors:** Adnan Qayyum, Hassan Ali, Massimo Caputo, Hunaid Vohra, Taofeek Akinosho, Sofiat Abioye, Ilhem Berrou, Paweł Capik, Junaid Qadir, Muhammad Bilal

**Affiliations:** 1 Information Technology University of the Punjab, Lahore, Pakistan; 2 NHS Bristol Heart Institute, University of Bristol, Bristol, UK; 3 University of the West of England, Bristol, UK; 4 College of Engineering, Qatar University, Doha, Qatar; 5 Birmingham City University, Birmingham, UK; 6 UNSW, Sydney, Australia

**Keywords:** Computer science, Biomedical engineering

## Abstract

Over the past few years, surgical data science has attracted substantial interest from the machine learning (ML) community. Various studies have demonstrated the efficacy of emerging ML techniques in analysing surgical data, particularly recordings of procedures, for digitising clinical and non-clinical functions like preoperative planning, context-aware decision-making, and operating skill assessment. However, this field is still in its infancy and lacks representative, well-annotated datasets for training robust models in intermediate ML tasks. Also, existing datasets suffer from inaccurate labels, hindering the development of reliable models. In this paper, we propose a systematic methodology for developing robust models for surgical tool classification using noisy endoscopic videos. Our methodology introduces two key innovations: (1) an intelligent active learning strategy for minimal dataset identification and label correction by human experts through collective intelligence; and (2) an assembling strategy for a student-teacher model-based self-training framework to achieve the robust classification of 14 surgical tools in a semi-supervised fashion. Furthermore, we employ strategies such as weighted data loaders and label smoothing to enable the models to learn difficult samples and address class imbalance issues. The proposed methodology achieves an average F1-score of 85.88% for the ensemble model-based self-training with class weights, and 80.88% without class weights for noisy tool labels. Also, our proposed method significantly outperforms existing approaches, which effectively demonstrates its effectiveness.

## Introduction

In recent years, surgical data science has emerged as a promising discipline within the field of surgical science, promoting the adoption of data-driven methods such as machine learning (ML) and deep learning (DL) techniques. These advanced approaches have been instrumental in enhancing surgical scene understanding and tackling a wide range of intermediate tasks in surgery, including object detection (tools, tasks, hands), surgical workflow analysis, and tissue segmentation for visual risk tracking^1^. Surgical tool classification plays a crucial role, enabling downstream applications such as preoperative planning, interoperative situational awareness and service audit^2^. Moreover, it can be used to develop an automated surgical skills assessment system that can provide objective feedback on the dexterity of the practitioner’s surgical procedures, which is crucial for continuous professional development^3^. However, despite the significant attention given to surgical tool classification, only a few studies have thoroughly analysed the robustness of DL approaches in the presence of issues such as class imbalance, and label noise.

**Figure 1:**
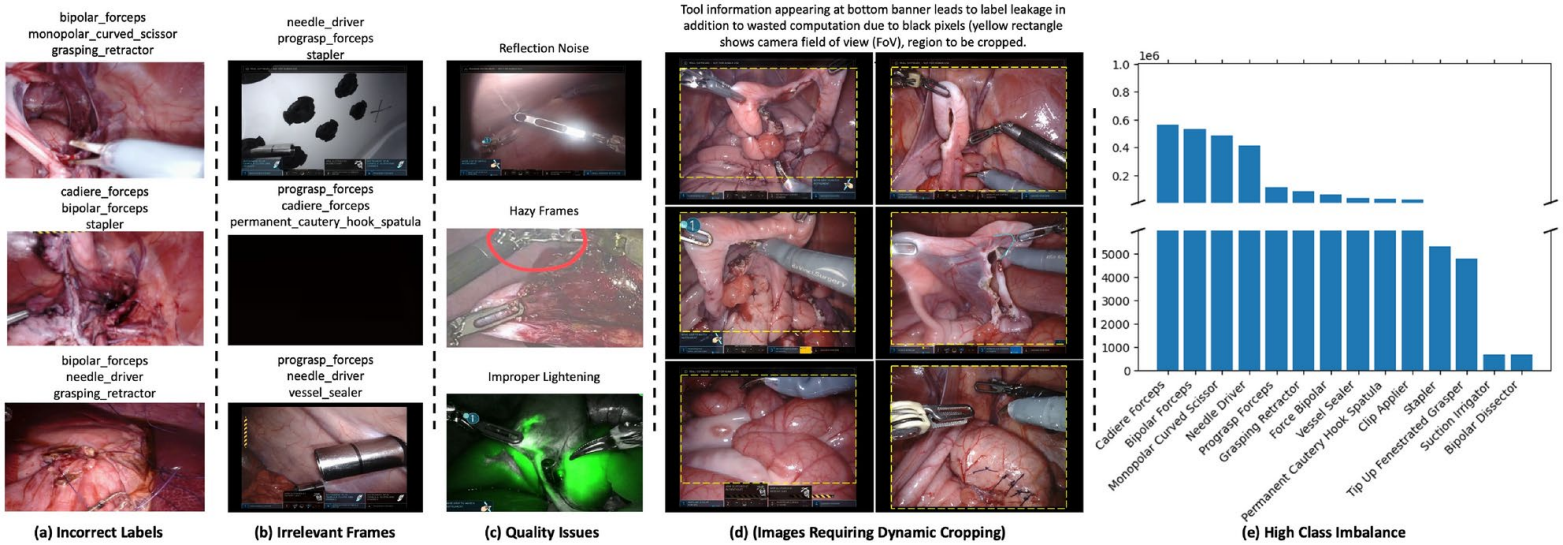
An illustration of surgical data quality issues in our dataset including incorrect labels, irrelevant frames, image quality issues, images leaking label information, and high class imabalance. Our proposed methodology is capable of training a robust model in the presence of these issues.

However, unlike other well-established disciplines, surgical data science is still in the developmental stage and lacks high-quality representative datasets necessary for developing robust applications to digitise clinical and non-clinical tasks^4^. Surgical datasets, such as endoscopic videos, commonly suffer from various data quality issues, including device-related noise, improper lighting, label leakage, class imbalance, and label noise (as shown in Fig. 1). Currently, most available datasets are insufficient for efficient and large-scale model training and contain imperfections. The creation of curated and well-annotated benchmark datasets for surgical tool classification poses significant challenges. Manually annotating surgical videos is exceptionally demanding due to factors such as limited expert availability, time/effort requirements, and wide diversity of surgical interventions and numerous tools across different procedures.

The development of robust models is imperative for successful translation of ML/DL-empowered products in clinical care^5^. To achieve state-of-the-art (SOTA) performance, DL models rely on large-scale clean data having high-quality annotations. However, annotating medical data poses significant challenges due to its costly and time-consuming nature^6^. Moreover, as previously discussed, surgical datasets frequently suffer from various data quality issues. These datasets often contain inaccurate labels with different types of noise, resulting in inaccurate depictions of surgical scenes. This issue is particularly prevalent in datasets acquired from emerging surgical robotic systems, where the camera feed is synchronised with the device log to generate labelled datasets. As surgeons manipulate the camera, tools may inadvertently fall out of focus while still attached to the robot, resulting in noisy labels in the log. To train robust models, a systematic approach is essential, incorporating diverse strategies for data preparation, model training, error analysis, and deployment on such noisy datasets.

In this paper, we aim to tackle the challenge of creating robust surgical tool classification models on challenging data characterised by high-class imbalance and significant label noise. We define robustness as the ability of the model to effectively learn relevant features from noisy data without significantly compromising its learning capabilities and predictive performance. Our dataset comprises approximately 24,694 30-second recordings of surgical robotic procedures, with one label per video indicating the presence of surgical tools. However, the surgical scene can undergo significant changes within a 30-second interval, resulting in substantial erroneous labels, particularly when video labels are extrapolated to frames extracted from these videos. Manual label correction for such a dataset, consisting of approximately 44.27 million frames, is impractical. To address these issues, we propose a novel active learning (AL) technique for efficiently labelling a minimal dataset. We then introduce an ensembling strategy for developing student-teacher models, enabling the learning of the underlying mapping function ($f: x \rightarrow y$) from the noisy data, where *x* and *y* denote input and output, respectively. To the best of our knowledge, this is the first study to comprehensively tackle these challenges by employing AL together with integrating ensembling in self-training to train a robust model using noisy data for surgical tool classification. This paper makes the following salient contributions.

1. We present a curated, well-annotated dataset comprising over 24k frames for 14 different surgical tools.
2. We propose an AL-based strategy to efficiently label surgical frames with minimal human effort.
3. We develop a student-teacher framework utilising an ensemble model, consisting of four DL models, for surgical tool classification using noisy data.
4. We employ weighted data loaders (WDLs) to train student models in a self-training framework, effectively addressing the high-class imbalance issue.
5. We perform a comprehensive analysis to validate the effectiveness of our framework, benchmarking both individual models and the ensemble model.

## Related work

Various approaches have been proposed for surgical tool classification, ranging from classical ML to DL-based solutions. Bouget et al.^7^ proposed a two-stage method that leverages the local appearance of surgical tools at the pixel level and enforces global shape using tool-specific shape templates. They emphasised importance of intermediate semantic labelling for achieving robust detection performance. Kumar et al.^8^ explored the use of classical image processing techniques, including point-based, region-based, and optical flow, for surgical tool detection and tracking. Richa et al.^9^ proposed a weighted mutual information-based image similarity function for visual tracking of surgical tools, specifically for proximity detection in retinal surgeries.

A number of existing research studies have used CNN for surgical tool detection. Jin et al.^3^ proposed a region-guided CNN model for surgical tool detection and tracking that was used for surgical skills assessment by evaluating tools’ movement, usage, range, and motion. Their method was the first attempt toward spatial localisation of surgical tools in laparoscopic surgical videos. Similarly, Liu et al.^2^ proposed a depth-wise separable convolution operation that was used to develop a convolutional LSTM model for surgical tool detection. The use of reinforcement learning to control positive and negative sample adaptation during the training model for surgical tool detection is presented in^10^. García-Peraza-Herrera et al.^11^ formulated the surgical tool detection problem as a segmentation problem and tracked it using optical flow. In their work, a fully convolutional network was used as the segmentation network; however, their method can distinguish between different tools.

In another work, Ciaparrone et al.^12^ employed the mask R-CNN model for the segmentation of surgical tools and evaluated 12 different backbone CNN architectures. In a similar study, Ceron et al.^13^ approached the surgical tool detection task as a segmentation problem and proposed a single-stage instance segmentation framework. Furthermore, they complemented the proposed segmentation model with a convolutional block-based attention mechanism, data augmentation, feature fusion, and anchor localization to enhance its performance. The proposed method was evaluated using the Robust Medical Instrument Segmentation 2019 challenge (ROBUST-MIS), where it achieved promising results even in the presence of various challenges such as smoke, occlusions, transparent tools, instrument flare, motion blur, etc. Twinanda et al.^14^ proposed a CNN model for surgical tool presence detection. The authors extracted features from a fully connected layer of the trained CNN model and then used the support vector machine model and the Hierarchical hidden Markov model for surgical phase detection. The proposed method was validated using two endoscopic surgical databases, i.e., Cholec80 and EndoVis. Hasan et al.^15^ proposed a novel framework named augmented reality tool network (ART-Net) for the detection, segmentation, and 3D rendering of surgical tools in endoscopic videos. The proposed framework is an integration of CNN architecture (with one encoder and multiple decoders) and algebraic geometry, which are collectively used to perform the aforementioned tasks. Shi et al.^16^ proposed a CNN-based framework that incorporates coarse and refined detection modules. Furthermore, they integrated an attention module into the refined detection module that enforces the network to learn important features for surgical tool detection. Yang et al.^17^ proposed a CNN-based model for surgical tool detection that works by generating ghost feature maps by exploiting intrinsic feature maps.

To address the problem of data imbalance, Jaafari et al.^18^ employed different data augmentation techniques (such as rotation of different angles, mirroring, shearing, and padding) while preserving the tool’s presence. The authors then fine-tuned a CNN (i.e., inception ResNet V2 pre-trained on ImageNet dataset) model using augmented data for the surgical tool classification task. To address the challenge of the availability of annotated data for surgical tool detection, Ali et al.^19^ presented a student-teacher-based self-supervised learning framework that works by only utilising a small fraction of labelled data. Labelled data is used for training the teacher model, which is then inferred using unlabeled images to get pseudo labels for student model training. In addition, the authors integrated a region proposal network for the extraction of the region of interest in input images. A weakly supervised framework named pseudo-supervised surgical tool detection (PSTD) that incorporates three phases for pseudo-label generation is presented in^20^. Specifically, to model the contextual information, PSTD employs a bi-directional adaptation weighting mechanism in the surgical tool detection classifier.

A novel modulated anchor network that works in conjunction with the Faster R-CNN model for surgical tool detection is presented in^21^. The key purpose of the anchor network is to predict the spatial location of anchor shapes used in tools for training the backbone network. Furthermore, they proposed to incorporate a relation module in the network (one module after each fully connected layer) to model the relationship of a tool in a given image with other tools. To address the limitations of anchor-based methods (i.e., handling variations in tool appearance), Loza et al.^22^ presented the use of a transformer network for surgical tool detection using multi-scale features. The employed transformer architecture uses positional encoding that helps capture the context and structural information of surgical tools of different sizes. Choi et al.^23^ present the utilisation of a SOTA object detection model, specifically You Only Look Once (YOLO), for surgical tool detection. However, only three surgical videos acquired at 25 fps were used for the evaluation of the model. We refer interested readers to comprehensive surveys that are focused on surgical tool detection for getting more detailed information about different DL-based methods^24–26^.

Ensembling has been found to be significant for surgical tool detection, as highlighted in various studies. For example, Alshirbaji et al.^27^ proposed an ensemble model that uses VGG16 and ResNet50 models for spatial feature learning and two LSTM units on top of CNN models for temporal feature learning. Similarly, Mishra et al.^28^ suggested a combination of CNN and stacked LSTM for spatial feature extraction and temporal information encoding. Jaafari et al.^29^, formulated surgical tool classification as a multi-label classification problem. To address this, they utilised an ensemble model comprising three CNN architectures: Inception v-4, VGG-19, and NASNet-A. They used various data augmentation techniques to address the data imbalance problem and improve model training. The dataset in this study comprises only 80 videos acquired at a frame rate of 25 and has seven labels. Wang et al.^30^ also formulated surgical tool classification as a multi-label classification problem and proposed an ensemble model that uses model averaging to ensemble predictions of trained GoogleNet and VGGNet. Our approach is similar to Jaafari et al.^29^ and Wang et al.^30^, as they have also used ensembling. However, our study differs in the following ways: (1) our dataset comprises more samples; (2) the number of classes (i.e., tools) in our data is 14; (3) we consider learning from noisy data; and (4) we incorporated ensembling in self-training. Aforementioned techniques could potentially be used for surgical tool classification task using the dataset employed in our study. However, it is imperative to note that these approaches may not be well-suited to address the distinctive challenges inherent in this dataset such as the presence of significant noisy labels, high class imbalance, and label leakage. This necessitate the development of a systematic approach for efficient model training even in the presence of such data imperfections. In the next section, we will present a systematic approach that includes active learning for creating clean labelled data with minimal manual efforts, segmentation-based dynamic cropping to avoid label leakage, student-teacher-based self-training to leverage unlabeled data while mitigating label noise, and weighted data loaders to address class imbalance issue.

## Methodology

In this section, we present our proposed methodology for surgical tool classification using noisy data. Specifically, we start by first defining the problem and describing the dataset.

### Problem formulation

Our objective is to develop a robust model for surgical tool classification using endoscopic surgical video (ESV) images, despite the presence of data imperfections and noisy labels. ESV data obtained from robotic systems often exhibit varying tool visibility within the field of view (FoV) throughout the duration of surgery. The process of annotating frames extracted from the ESV required extrapolating video labels, which introduced inconsistencies between ground truth labels and actual tools present in the view, leading to significant label noise. This noise presents a unique challenge for deep neural networks (DNNs) in accurately classifying tools in the image. Our objective is to address this challenge by predicting the presence of tools in each frame $x_i$. We formulate the surgical tool presence detection task as a multi-label image classification problem, aiming to classify surgical tools present in each frame.

We extract our dataset *D* from a collection of ESV clips comprising different minimally invasive surgeries performed by surgeons using da Vinci surgical robot. Each clip $c_j$ in the ESV collection consists of multiple frames $c_j = \bigcup _{i=0}^{f_j-1} \{x_j^{(i)}\}$ acquired at a frame rate of 60 frames per second (fps), where $f_j$ denotes the total number of frames in $c_j$. Furthermore, each ESV clip has been assigned a set of three unique labels $y_j = \{y_{j_1}, y_{j_2}, y_{j_3}\}$ by the dataset providers. Each of the three labels is an instance of the label set $L = \bigcup _{i=0}^{T-1} \{t_i\}$, where $T=14$ denotes the total number of tools (i.e., classes) present in the dataset. Therefore, our dataset can be represented as ${D} = \bigcup _{j=0}^{n-1} \{(c_j, y_j)\} = \bigcup _{j=0}^{n-1} \bigcup _{i=0}^{f_j-1} \{(x^{(i)}_j, y_j)\}$, where $n=24,694$ is the total number of ESV clips. For simplicity, we denote our dataset as ${D} = \bigcup _{i=0}^{N-1} \{(x_i, y_i)\}$, where is $x_i \in {R}^{1280\times 720\times 3}$ represents a single frame and $l_i \in L$ represents the set of labels assigned to the ESV clip from which $x_i$ is sampled. Here, *N* denotes the dataset size.

### Data description and preprocessing

#### Data description

The dataset used in this study is sourced from the “Surgical Tool Localisation in Endoscopic Videos” challenge held at the Medical Image Computing and Computer Assisted Intervention (MICCAI) 2022 conference^31^. It consists of 24,694 ESV clips captured during surgical training exercises using the da Vinci robot. These videos are recorded at a 60 frames per second (fps) rate with a resolution of 720p ($1280\times 720$). This dataset requires  15.4 terabyte of disc space if we completely extract its frames. Each ESV clip is accompanied by corresponding tool presence labels. However, it’s worth noting that instances exist within the dataset where the ESV label indicates the presence of tools not visible in the video due to surgeons moving tools out of the field of view (FoV), despite their installation on the robotic system. Consequently, noise is introduced when generating ESV clip labels by extracting tool information directly from the robotic system. Furthermore, the dataset presents various data quality issues (see Fig. 1), including weak labels, blank frames, and label leakages. Label leakage occurs when the user interface of the robotic system, visible at the bottom of ESV images, reveals the names of deployed tools, further complicating the modelling task. The challenge of inaccurate labels is compounded when weak ESV labels are extrapolated across frames, resulting in substantial label noise in the dataset. This presents a significant hurdle for models in learning robust features for surgical tool classification. Figure 1e illustrates the distribution of the fourteen surgical tools within the dataset, highlighting its highly imbalanced nature, which poses an additional challenge in training a robust model for surgical tool classification.

#### Data preprocessing

In our proposed method, we conducted thorough data preprocessing to tackle various data quality-related issues. We present a systematic methodology for training robust models from datasets containing significant label noise. Next, we delve into the techniques employed for preprocessing videos and labels, which form the core of our proposed methodology.

**(a) Videos Preprocessing:** The following are the key steps involved in preprocessing of ESV clips.

*Frame Sampling:* Our initial challenge involved sampling a minimal number of frames from the ESV clips to conduct experiments efficiently within a reasonable timeframe. With an average of 1800 frames per ESV clip, the total exceeds 44.27 million images, with each frame $x_i \in {R}^{1280\times 720\times 3}$ occupying over 15.42 TB of storage space on the hard drive if extracted as JPEG files due to compression. Utilising all frames from such an extensive imaging dataset for training models is computationally infeasible. Furthermore, the surgical scene undergoes minimal changes between consecutive frames, rendering many frames useless for learning additional meaningful features by the models. To address this issue, we first compressed the ESV clips to a frame rate of 10Hz. Subsequently, we employed OpenCV to extract approximately twenty keyframes from each ESV video, ensuring each keyframe captured a significant scene difference compared to its preceding frames. This approach provided an adequate number of samples for model training and enabled the execution of our experiments within a reasonable timeframe.

*Dynamic Region Cropping using Segmentation Model:* Our preliminary analysis revealed that models struggle to learn robust features for classifying surgical tools due to label leakage, where tool information from the robotic system’s UI banner is inadvertently included in the frames. This issue led the model to take shortcuts instead of focusing on learning meaningful classification features^32^. To address this, we trained our own segmentation models to dynamically crop out pixels containing leaked tool information. We randomly sampled one frame per ESV clip and used the Prodigy annotation tool to create a small segmentation dataset of masks to differentiate between foreground and background image regions. The foreground region contains useful surgical scene information, while the background region comprises irrelevant pixels, revealing tool information. We trained a U-Net-based segmentation model to segment the foreground region in the frames. Subsequently, we used OpenCV to crop the entire dataset, effectively discarding the leaky regions. Our segmentation model efficiently eliminates the UI control panel at the bottom of all frames, the disclaimer notice at the top, and the black borders on the left and right sides of the videos (see Fig. 1d). These additional pixels not only contribute to prolonged network training and wasted computation but also significantly impact the learning algorithm’s ability to learn robust classification features^33^. For more details about our dynamic segmentation model and results, please refer to our published work on addressing label leakage in ESV^34^.

*Black Frames Removal:* Some ESV clips consist entirely of black frames devoid of any visual information, while others contain partially black frames. Despite the lack of visual content, these frames still received tool labels due to label extrapolation from the corresponding ESV clip. Such occurrences typically coincide with the installation or removal of the camera lens for cleansing or redeployment on another arm. Initially, we opted to remove these frames entirely from the dataset. However, we later decided to address this issue by introducing a ‘blank‘ class and augmenting the dataset with several black images. This allowed the models to accurately recognise and classify black frames in ESV clips, leading to improved predictive performance on the validation set.

**(b) Labels Preprocessing:** We performed exploratory data analysis to find some prominent labelling noise in the dataset and fixed the identified issues using the following methods.

*Labels Standardisation:* We used regular expressions to standardise tool labels. This involved removing various symbols such as brackets ([]), quotes (”), hyphens (-), slashes ($\backslash$ $\backslash$). Also, the underscore (_) symbol was removed with a white space character.

*Labels Extrapolation:* In the dataset, each ESV clip is provided with a single label string describing tools presence. As described earlier, we extract keyframes from these ESV clips and then extrapolate the corresponding ESV label to these keyframes. However, this extrapolation introduces further noise into the dataset. Many frames are created where the label indicates the presence of three instruments, yet only two or fewer tools are visible in the given frame. This scenario often occurs when the surgeon moves a tool out of view, despite its installation on the robotic system. Learning from noisy labels is recognised as one of the unique challenges presented in this competition.

### Training baseline model(s)

To facilitate AL in our proposed methodology, we trained a baseline model for surgical tool classification using noisy dataset. The primary objective of the baseline model is to identify samples in the dataset for manual labelling based on model loss/uncertainty on surgical tool classification task. It also laid the groundwork for extensive experimentation to devise a robust ML training pipeline to ensure the development of improved models in subsequent phases. In machine learning practice, the importance of employing data augmentations for training superior deep learning models is widely acknowledged. With this principle in mind, we explored various data augmentation approaches to preprocess batches while training our models. Specifically, we tested three techniques: squishing, cropping, and padding. Our findings revealed that squishing images yielded superior results compared to cropping and padding. Furthermore, we investigated the impact of rectangular versus squared resising. We found that baseline models achieved better performance when the dataset was scaled to maintain a similar aspect ratio as the original dataset, which had an original rectangular dimension of 1280$\times$720. This insight guided our modeling choice to preserve the aspect ratio during the process, aiming for optimal model performance.

*Ensemble model *$E(\cdot )$: We employed ensembling to train baseline, teacher, and student models. Our ensemble model $E(\cdot )$ comprises four component models from the following model families: $M_c(\cdot )$, representing ConvNext^35^; $M_v(\cdot )$, corresponding to ViT^36^; $M_s(\cdot )$, representing Swin V2^37^; and $M_r(\cdot )$, based on RegNextX^38^. We carefully selected variants from these families after thorough experimentation using grid search optimisation for architectural search to design ensemble learners. Specifically, we explored different neural network architectures, including both smaller and larger networks. The smaller variants include convnext_small_in22k, regnetx_016, vit_small_patch16_224, and swinv2_base_window12_192_22k, while the larger variants are convnext_large_in22k, regnetx_320, vit_large_patch16_224, and swinv2_large_window12_192_22k. We conducted exhaustive experimentation to determine which model families and architectural combinations could learn the given task with greater accuracy. Additionally, ViTs and RegNetX showed promising performance. Upon evaluation, we observed that the larger architectural variants did not yield noticeable performance improvements; instead, they exhibited significant overfitting. Consequently, we opted to utilise the smaller architecture variants for our ensemble.

*Ensemble loss *${L}_z(\cdot )$: We then use *D* to train $E(\cdot )$ using an ensemble loss function ${L}_z(\cdot )$, defined below:

1 $$\begin{aligned} {L}_z ( E(x), y ) = \alpha {L} ( M_c(x), y ) + \beta {L} ( M_v(x), y ) + \gamma {L} ( M_s(x), y ) + \delta {L} ( M_r(x), y ) \end{aligned}$$

where *x*, *y* denote the input and the corresponding ground truth, respectively and ${L}(\cdot )$ denotes the standard multi-class classification loss of the DNN over *D*, respectively, and $\alpha , \beta , \gamma$ and $\delta$ are the regularization hyperparameters tuned based on the predictive confidence (loss) of each model.

### Proposed ensemble based self-training method

**Figure 2:**
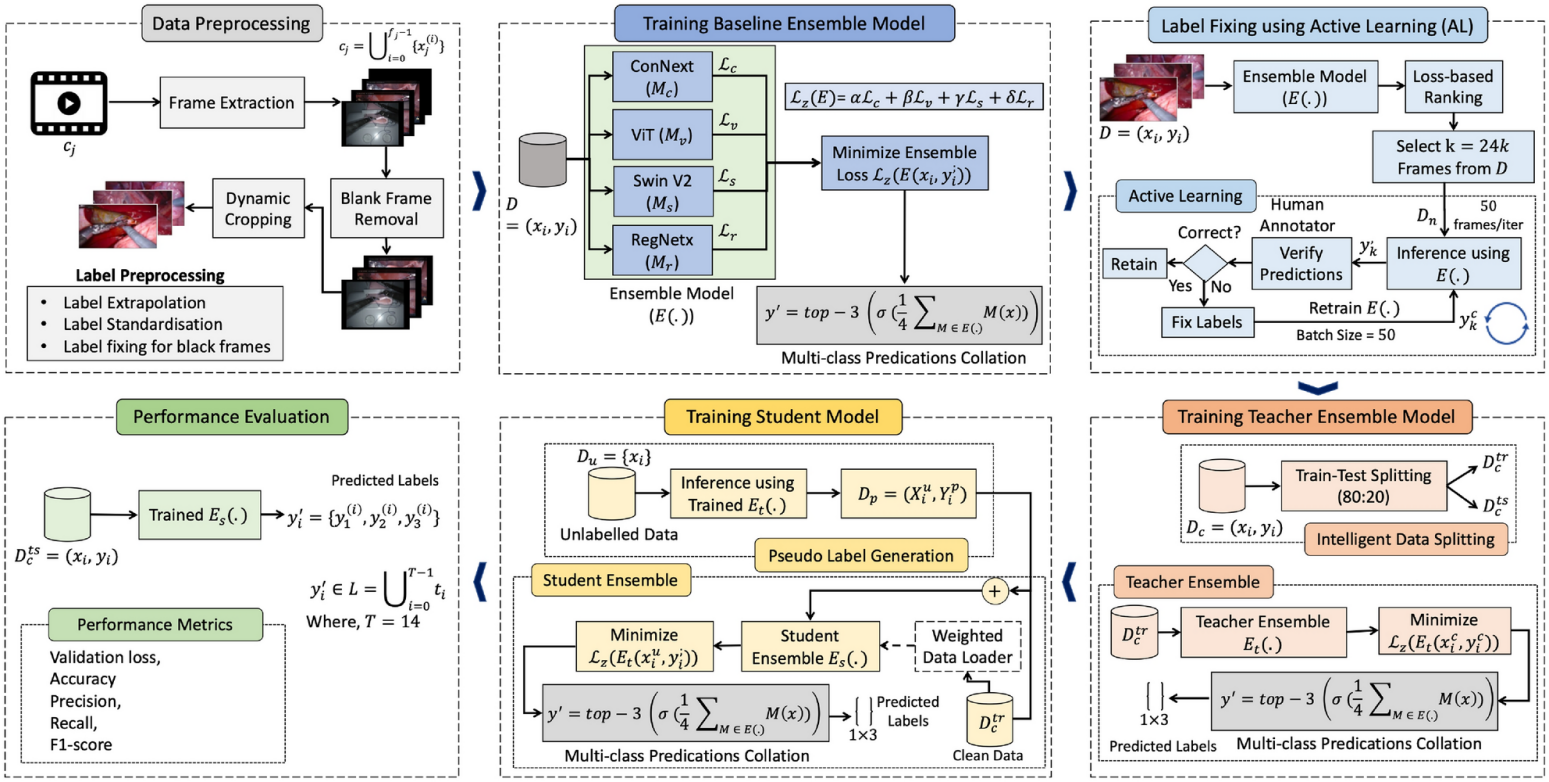
Proposed methodology for robust surgical tool detection. *First,* we address data imperfections through preprocessing. *Next,* we train a baseline ensemble model, followed by loss-based epistemic scoring for minimal dataset selection to fix labels. *Then,* we employ active learning for manual label correction. *Afterwards,* we train a teacher ensemble model $E_t(\cdot )$ to generate pseudo labels. *Subsequently,* these pseudo labels are utilised to train the student ensemble model $E_s(\cdot )$. *Finally,* we assess the performance of the proposed student ensemble model.

In this section, we will discuss our proposed self-training (student-teacher model learning)-based strategy for the robust detection of surgical tools using noisy labels. Our proposed ensemble learning approach for surgical tool classification using self-training is presented in Fig. 2. The following three key steps are involved in the proposed self-training framework: (1) creating clean (human-)labeled data using active learning; (2) training student-teacher ensemble models using a self-supervised learning strategy; and (3) using WDLs to ensure fair learning and address high-class imbalance issues, described next.

#### Label cleaning using active learning

To quantify the extent of label noise in our ESV data *D*, we first perform an extensive exploratory data analysis. Our analysis revealed that the original video-level labels also contained significant label noise, which was further amplified after the extrapolation of video-level labels to frame-level. To address this issue, we propose to leverage AL to create clean data ${D}_c$ with minimal manual effort, where $|{D}_c| \ll |{D}|$. Note that ${D}_c$ is sampled from noisy data *D* for fixing incorrect labels through an iterative AL process that encompasses three major steps. *In the first step*, we train a baseline ensemble AL model $E(\cdot )$ using *D* and then used the trained $E(\cdot )$ to automatically identify and manually fix potentially mislabeled input samples to create ${D}_c$. *In the second step*, we identify a set of $k=24997$ samples from *D* on which the ensemble model $E(\cdot )$ exhibits the highest classification error (signifying that $E(\cdot )$ finds it hard to accurately predict these frames due to the presence of label noise), where $k \ll N$. Formally, the process of identifying these samples can be expressed as:

2 $$\begin{aligned} \text {Repeat { k} times}: {D}_c = {D}_c \cup \underset{x_i \in {D}, x_i \notin {D}_c}{{\text {argmax}}} {L}_z( E(x_i), y_i ). \end{aligned}$$

For each AL iteration, we compute the output of $E(\cdot )$, which is a set of the maximum probability assigned to each class $t_i \in L$ by any model $M \in E(\cdot )$,

3 $$\begin{aligned} E(x) = \bigcup _{t_i \in L} \max _{M \in E} \{ M (t_i | x) \}, \end{aligned}$$

where *L* denotes the label set (as defined previously). *In the third step*, we then ask two human experts to verify the model predictions in each iteration of AL, which uses a batch size of 50 frames (for manually label fixing and fine-tuning $E(\cdot )$ for each AL iteration). In case the experts identify model predictions as incorrect, they manually suggest correct labels for that particular frame and the corrected frame-labels pair is then used to update ${D}_c$. To minimise human interventions in fixing incorrect labels, we fine-tune the underlying $E(\cdot )$ when a complete batch has been verified manually. This strategy resulted in the reduction of manual efforts as the AL process progressed (empirical results will be presented in the next section). Note that initially, manual annotations were done by our clinical partners and then a team of two annotators was trained by clinical experts to perform remaining annotations. Finally, to ensure the correctness of clean data ${D}_c$ and to eliminate or fix any discrepancies, all annotations were validated by clinical experts. The aforementioned AL process is repeated several times to let our active learning ($E(\cdot )$) continue to improve its performance on hard samples as the noisy labels get fixed, until the size of ${D}_c$ becomes *k*. Finally, the compiled clean data ${D}_c$ is divided into two distinct non-overlapping sets: the clean training data ${D}_c^{tr}$ (comprising 80% of ${D}_c$) and the clean test data ${D}_c^{ts}$ (comprising the remaining 20% of ${D}_c$).

#### Student-teacher formulation for ensemble learning

We proposed to leverage student-teacher formulation (i.e., self-supervised learning also known as self-training) to automatically mitigate the effect of noisy labels in the surgical tool classification task. Our proposed ensemble learning approach for surgical tool classification using self-training (i.e., student-teacher model) works in two steps, as presented in Fig. 2.

*In the first step*, we train teacher ensemble model $E_t(\cdot )$ having same architecture as $E(\cdot )$ defined previously. We train $E_t(\cdot )$ over ${D}_c^{tr}$ using ${L}_z(\cdot )$, and evaluate $E_t(\cdot )$ over ${D}_c^v$. Given an input *x*, the output of $E_t(\cdot )$ is calculated as sigmoid of mean non-probabilistic outputs (logits) of all models $M \in E_t(\cdot )$.

4 $$\begin{aligned} E_t(x) = \sigma \left( \frac{1}{4} \sum _{M \in E_t(\cdot )} M(x) \right) \end{aligned}$$

*In the second step*, we re-label the unclean data $({D} - {D}_c)$ by first querying $E_t(\cdot )$ with $({D} - {D}_c)$ to get the pseudo labels $E_t(x), \forall x \in ({D} - {D}_c)$, and use these pseudo labels to create a pseudo labelled dataset ${D}_s = \{(x_i, E_t(x_i))\}, \forall x \in ({D} - {D}_c)$. Finally, we define a student ensemble model $E_s(\cdot )$ (having same architecture as $E(\cdot )$), and train $E_s(\cdot )$ over the augmented dataset ${D}_s \cup {D}^{tr}_c$. As before, the output of $E_s(\cdot )$ at the inference time is computed by passing the average non-probabilistic outputs of all models $M \in E_s(\cdot )$ through the sigmoid function and using the top-3 labels as the final classification decision.

#### Augmenting self-training-based ensemble learning

In addition to the noisy labels, our dataset also suffers from significant class imbalance issues (as evident from Fig. 1e). To overcome this issue we proposed to use WDLs to train the ML models while reducing the data bias due to class imbalance. We fine-tuned the ensemble models for several epochs using WDLs to improve performance. Also, to further augment the capacity of our self-training-based ensemble model in learning hard labels, we employed label smoothing, which is a widely used regularisation technique to improve model generalisability and prevent overfitting. Label smoothing defines a soft distribution over classes instead of using hard targets, i.e., it allows models to relax label boundaries slightly. Therefore, instead of 1 s and 0 s, the loss function is engineered to use a number less than 1 for 1s and a number a bit more than 0 for all 0 s in the encoding vector. In this way, label smoothing assigns probability *p* to a correct class and uniformly distributes the remaining ($1-p$) to the rest of the classes. This enables the model to be more robust to the perturbations in the input while sacrificing a bit of predictive confidence. In the literature, label smoothing has been shown quite successful in improving the performance and generalisability of DL models using noisy datasets^39^. The algorithm for our proposed methodology is described in Algorithm 1.

5 $$\begin{aligned} E_s(x) = {\text {top-3}} \left( \sigma \left( \frac{1}{4} \sum _{M \in E_s(\cdot )} M(x) \right) \right) \end{aligned}$$

**Algorithm 1:**
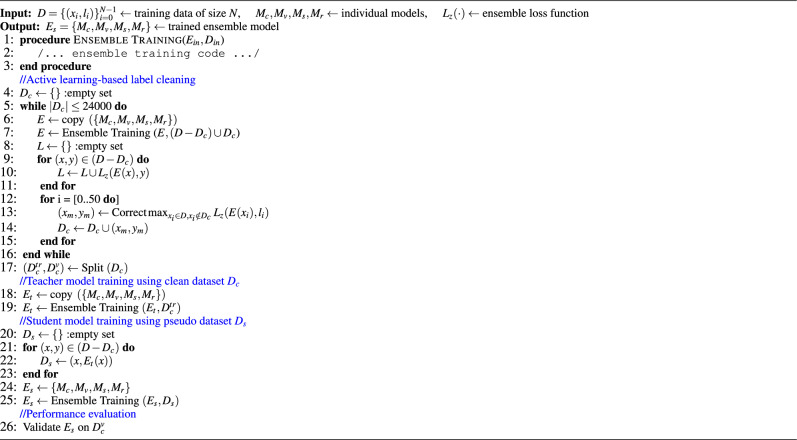
Methodology

## Results and discussions

### Experimental setup

*Stratified Data Splitting:* We encountered challenges with random allocation of images into training and test sets, which could lead to label leakage and imbalanced tool labels. To address these issues, we implemented a novel stratified sampling approach. Initially, we clustered ESV clips based on unique label combinations and then evenly distributed these images across the two sets based on the split ratio. This method ensures that each tool combination is represented in both training and testing sets, facilitating fair model validation. Notably, our dataset includes eight tool combinations, each with only one video, exempting this policy. Employing this strategy, we divided our dataset into training and testing sets with an 80% and 20% split, respectively.

*Hyperparameters Selection:* An exhaustive hyperparameter tuning process was conducted in training models using the W &B library, taking into account data augmentations, batch sizes, learning rates, and architecture choices. Through experimentation, it was determined that image squishing provided better results compared to cropping. Furthermore, the cyclic learning rate approach, as introduced by Smith et al.^40^, was adopted for selecting the best learning rate schedule, combining stochastic gradient descent with warm restarts, allowing for an annealing schedule with periodic restarts. Among the different learning rates tested, a value of 1e-2 consistently yielded the best performance across the ensemble models. To facilitate efficient batch processing, a batch size of 64 was utilised, with n_workers set to 8 for parallel processing. Additionally, mixed precision training (FP16) was employed, resulting in a 20% improvement in computation time. The training process involved initially training the models’ heads using the fit-one-cycle for 12 epochs. Subsequently, the entire model was unfrosen, and training was continued for an additional 12 epochs using smaller learning rates, such as slice (1e-2/400, 1e-2/4). This progressive training strategy allowed the models to refine their performance by focusing on fine-tuning the lower layers while maintaining the learned features in the higher layers. To ensure a fair comparison, all models were trained using the same choice of hyperparameters.

*Performance Metrics*: Our surgical tool classification task involves multi-label classification, aiming to accurately identify the presence of surgical tools among fourteen options in each ESV frame. To evaluate the models’ performance effectively, we employed established metrics for multi-class classification, including accuracy, precision, recall, and f1-score. Additionally, we introduced aggregated performance measures to ensure a comprehensive evaluation across diverse samples and tool categories. These include mean average accuracy (mAA), mean average recall (mAR), mean average precision (mAP), and mean average f1-score (mAF1). It’s important to clarify that these metrics are tailored specifically for classification tasks. The inclusion of ’mAP’ in our terminology aims to maintain consistency in reporting results across different metrics, although it differs from the mAP metric commonly used in object detection models.

### Baseline results

To establish a baseline for our experiments, we initially divided our original noisy dataset, *D*, into training set $D^{tr}$ and testing set $D^{ts}$, using an 80% - 20% split, respectively. We then conducted fully supervised training of our baseline models, which included convnext_small_in22k, regnetx_016, vit_small_patch16_224, and swinv2_base_window12_192_22k, along with an ensemble model, for the task of surgical tool classification using $D^{tr}$. Following training, we evaluated the models on the test data, $D^{ts}$. Table 1 presents an overview of the baseline results, detailing various performance metrics such as accuracy, precision, recall, and F1-score. Notably, our proposed ensemble model outperformed the individual models in terms of accuracy (99.9%) and precision (98.4%). However, it’s important to mention that the convnext_small_in22k model exhibited relatively lower performance compared to the other models (regnetx_016, vit_small_patch16_224, and swinv2_base_window12_192_22k), while these three models demonstrated similar performance across most metrics. It is worth noting that the results reported in Table 1 indicate high performance for the baseline models across different metrics (this is for the case when model are trained and evaluated using noisy data). However, further investigation reveals a different reality when these models are tested on cleaned data, where the results of this analysis are also reported in Table 1. It is evident that the performance of the baseline models significantly deteriorated, with mean F1 score of the proposed ensemble model dropping to 18.9%. The inability of baseline models to accurately capture robust features to maintain performance on cleaned data underscores the challenges posed by noisy labels.

**Table 1:** Baseline results for surgical tool classification using original (noisy) data.

Model	#Parameters	#Trainable params.	Training and testing using noisy data				Noisy training and testing using clean data			
			mAP	mAR	mAA	mAF1	mAP	mAR	mAA	mAF1
ConvNext	197.81M	1.67M	0.96628	0.93587	0.99526	0.94968	0.1141	0.1072	0.7232	0.0775
RegnetX	107.75M	2.62M	0.97359	0.92547	0.99425	0.94676	0.1138	0.1048	0.7232	0.0773
VIT	303.66M	0.66M	0.97978	0.95983	0.99610	0.96920	0.1139	0.1048	0.7232	0.0773
Swin V2	66.47M	0.59M	0.97649	0.96780	0.99651	0.97196	0.1138	0.1047	0.7231	0.0772
Ensemble	675.69M	5.54M	0.98404	0.95724	0.99853	0.969401	0.2539	0.1871	0.8775	0.189

In our multi-class classification task, mean average precision (mAP) represents the average precision across various tool labels and frames used for evaluation.

### Results for label cleaning using AL

**Figure 3:**
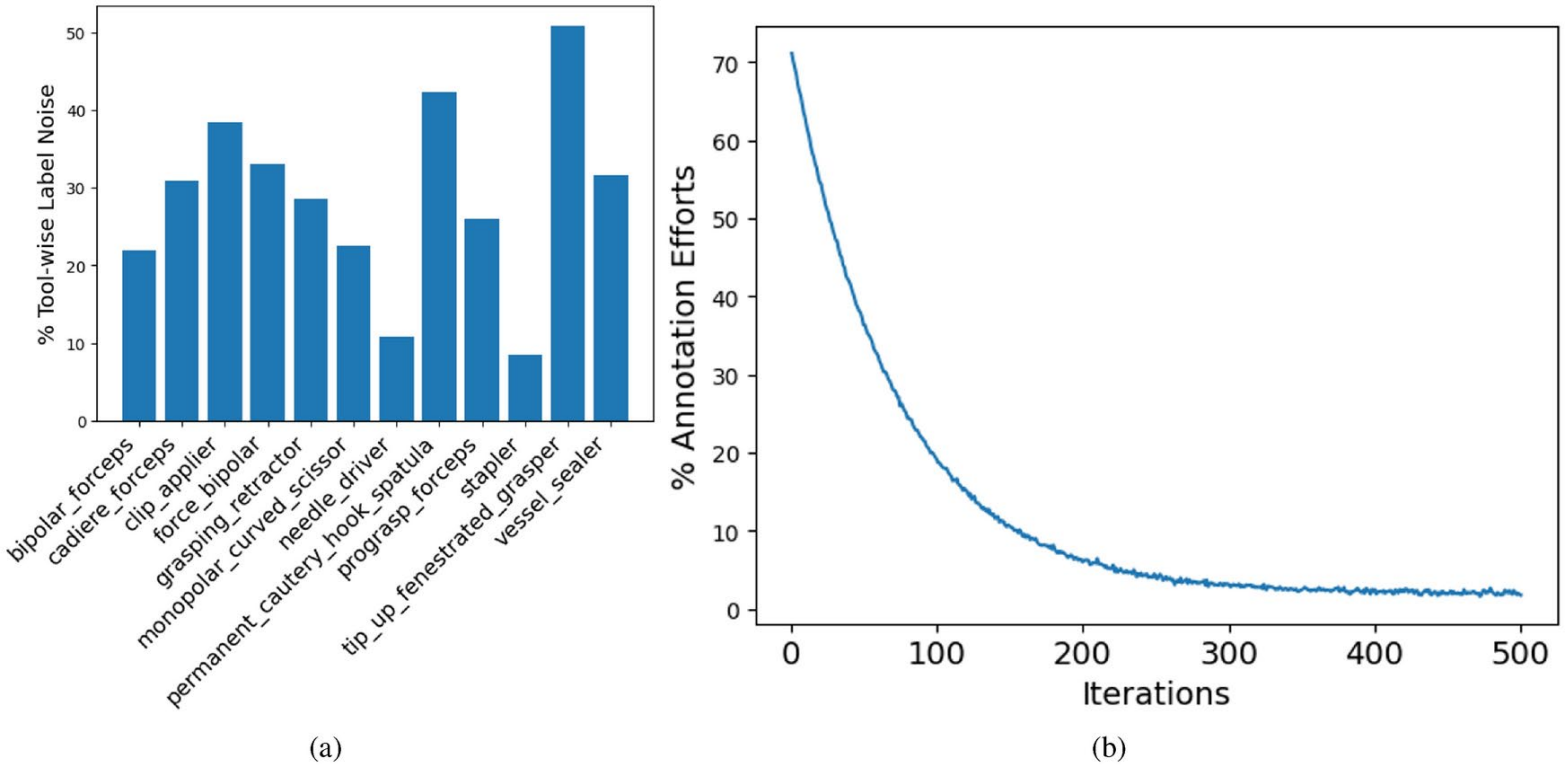
Demonstrating the efficacy of AL-based manual data annotation with minimal human effort. Figure 3a illustrates the percentage of required label corrections for each tool category due to inaccurate labels, while Fig. 3b highlights the effectiveness of our weakly-supervised AL-based annotation strategy in reducing manual label corrections over iterations. For instance, initially, 70% of frames in a batch required manual label corrections, but as the AL process progressed, this manual effort was reduced to only a few samples.

To address the challenge of significant label noise, we employed a label-cleansing strategy. Given the impracticality of manually reviewing and rectifying labels for the entire dataset due to its large size, we employed a loss-based epistemic scoring method to identify a minimal subset (5%) of the dataset for manual labelling. Our manual labelling process was facilitated by an AL-based semi-automated annotation strategy. We leveraged the Prodigy tool to implement our AL strategy. In the AL framework, human experts initially manually corrected the tool labels for the first batch of samples. This triggered the retraining of the baseline ensemble learner using the corrected samples, resulting in relatively accurate default labels for subsequent batches. Each time a batch was processed, the retraining process was automatically initiated, progressively improving the model’s performance. Figure 3a provides insights into the proportion of noise present for each surgical tool that required correction during the labelling process. Tool-wise label noise is calculated using a comparative analysis of labels from AL-assisted cleaned data to that with the labels of original (noisy) data. Furthermore, it is worth noting that the label noise shown in Fig. 3a serves as an estimate rather than an exact representation of the true label noise, which in reality will be significant. As the estimated label noise presented in Fig. 3a only accounts for the significantly reduced size of the clean labelled dataset, which comprises only 10% of the original noisy data. Additionally, we present an analysis based on the number of label corrections required through manual intervention in Fig. 3b. It is evident that as our label cleansing strategy advanced and we continued fine-tuning our ensemble model, the number of manual corrections required significantly decreased, reducing the workload for human experts through AL.

**Figure 4:**
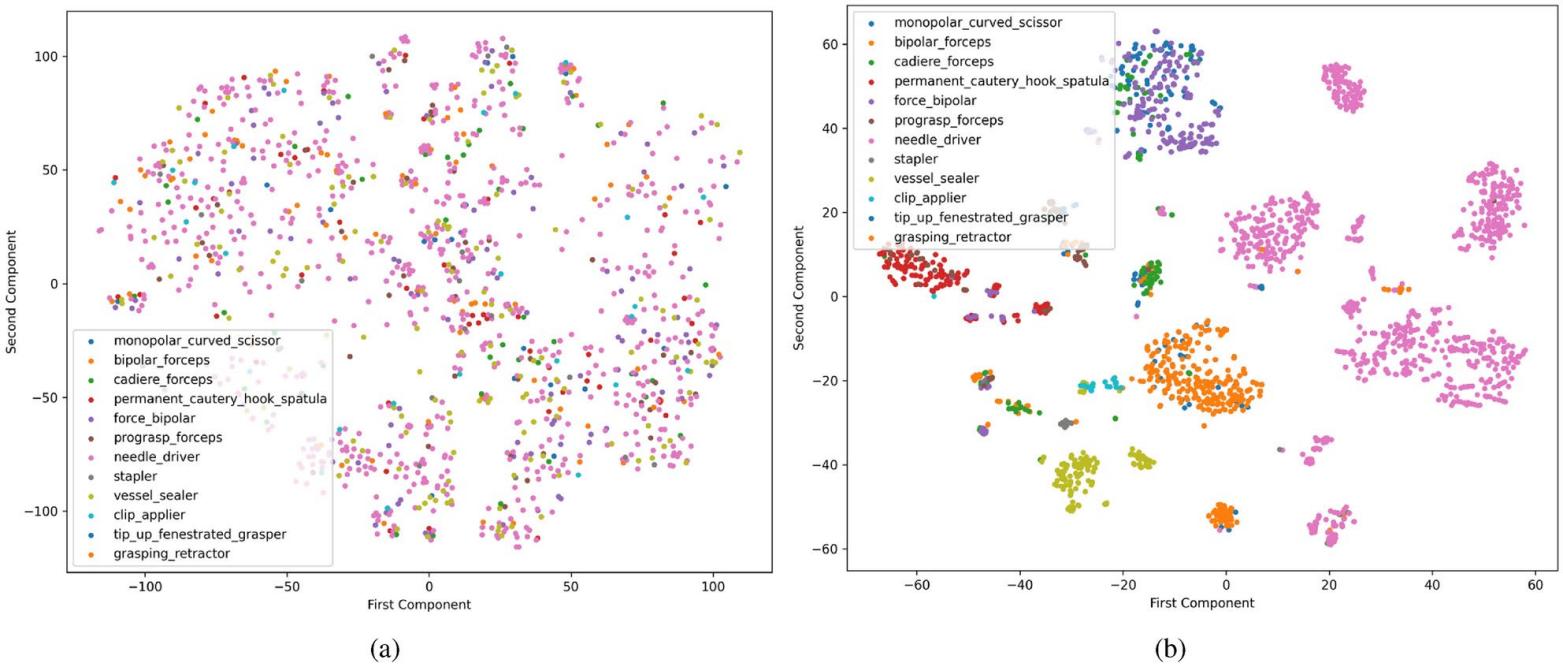
Illustrating the effect of noisy labels on the learned embeddings using t-SNE. (**a**) Demonstrates the embeddings extracted from the ConvNext model trained using noisy data, while (**b**) depicts the embeddings extracted from the ConvNext model trained using clean data.

To demonstrate the impact of noisy labels on the model’s learning capabilities, we extracted embeddings from models trained under two conditions: one using noisy data and the other using clean data. These embeddings were then visualised in a two-dimensional space using t-distributed Stochastic Neighbour Embedding (t-SNE), which is a widely used technique that effectively captures latent patterns from high-dimensional data. The results of this analysis are presented in Fig. 4, which demonstrates significant differences among t-SNE visualisation of embeddings extracted from the model trained on noisy data (Fig. 4a) and learned embeddings from a model trained using clean data (Fig. 4b). For instance, it is evident from Fig. 4a that data samples are dispersed, indicating poor class separability and substantial overlap among different categories. This dispersion suggests that the model struggles to discern distinct classes, likely due to the confounding influence of label noise during training. Conversely, embeddings from the model trained on clean data are seen to create well-defined and segregated clusters for different classes with minimal overlap between them. Such distinct clustering indicates that the model has successfully learned to differentiate between classes, reflecting the efficacy of training with high-quality noise-free data. This comparative analysis underscores the detrimental effects of noisy data on the learning process and the model’s capability to develop robust and discriminative representations. It further reinforces the necessity for accurately labelled data to train reliable and effective models in practical applications. Furthermore, to provide insights into the class-wise performance of surgical tool classification, we measure different performances for each tool. The results for this analysis are presented in Table 2, which suggests that our proposed technique was able to effectively classify different tools irrespective of high-class imbalance.

**Table 2:** Statistical results in terms of different performance metrics for each surgical tool.

Tool name	Samples	Precision	Recall	F1-Score
Needle_driver	1280	0.95	1	0.97
Monopolar_curved_scissor	949	0.98	0.99	0.98
Force_bipolar	320	0.91	0.83	0.87
Clip_applier	36	0.55	0.62	0.58
Tip_up_fenestrated_grasper	9	0.26	1	0.42
Cadiere_forceps	585	0.88	0.86	0.87
Bipolar_forceps	786	0.97	0.94	0.95
Vessel_sealer	281	0.84	0.89	0.86
Prograsp_forceps	398	0.81	0.79	0.8
Stapler	38	0.69	0.5	0.58
Permanent_cautery_hook_spatula	276	0.96	0.99	0.97
Grasping_retractor	42	0.53	0.76	0.63

### Self-training-based ensemble model results

In this section, we present the results of our proposed ensemble model-based self-training approach, which involves training two sets of models: a teacher model and a student model. The teacher model ${M}_t$ was trained using clean data obtained using the AL strategy. The purpose of the ${M}_t$ was to infer new (pseudo) labels from the model and discard original noisy tool labels. This pseudo-labelled dataset is used to train ${M}_s$ and validate using the clean AL labels data. ${M}_t$ builds upon the models trained in the AL, while the student model ${M}_s$ further extends ${M}_t$ through transfer learning. The results of the student-teacher model-based surgical tool classification in terms of various performance metrics, are summarised in Table 3. The table presents the performance of the proposed ensemble model as well as the individual models trained at this stage. It is evident that the ensemble strategy outperforms all individual models in both teacher and student settings. Notably, the results in Table 3 indicate a decline in performance metrics other than accuracy compared to our baseline results (reported in Table 1). This highlights the importance of considering metrics beyond accuracy alone in evaluating surgical tool classification models. Additionally, the table demonstrates that the proposed student ensemble model, in the self-training setting, performs comparatively better than the teacher model. This improvement is due to the generation of high-quality pseudo labels by ${M}_t$ (while eliminating label noise), despite the significant difference in the number of samples used for training teacher and student models.

#### Augmenting self-training using label smoothing

We trained models using hard labels, assigning a value of 0 to all surgical tools except those present in the image. This approach encouraged the models to predict activations with higher confidence levels, potentially leading to overfitting despite the lack of meaningful probabilities. Even when uncertain, the models would often assign a value of 1 to the predicted tool due to the influence of noisy data used as pseudo labels. While these pseudo labels were an improvement over the original data, they still contained imperfections. Compounding this challenge, certain tools in our dataset closely resembled each other when viewed from specific angles, such as monopolar_curved_scissor versus permanent_cautery_hook_spatula, clip_applier versus stapler, or bipolar_forceps versus bipolar_dissector. To address these issues, we explored the effectiveness of various regularisation techniques, including label smoothing, to improve the generalisability of our proposed ensemble model. Specifically, we implemented label smoothing using Fastai’s LabelSmoothingCrossEntropy loss function during training. The results of applying label smoothing to both standalone models and the ensemble model, trained using a student-teacher-based self-training approach, are summarised in Table 4. Notably, label smoothing was only applied during the training of the student models. Overall, label smoothing demonstrated promising results in mitigating the impact of noisy labels, albeit with a slight decrease in performance metrics. This decline in performance can be attributed to the relaxation of decision boundaries when transitioning from hard labels to soft labels, as discussed in the literature (Szegedy et al., 2016). From the results presented in Table 4, we observed that, on average, the ensemble model with label smoothing performed slightly worse compared to the other models. However, it’s important to highlight that the ensemble model consistently outperformed all other models in terms of the crucial F1-score metric. Additionally, Swin V2 exhibited superior performance in terms of accuracy, precision, and recall.

**Table 3:** Performance evaluation of four baseline models and ensemble models using a self-supervised learning approach (i.e., student-teacher model formulation), measured across various performance metrics.

Model type	Labelling strategy	Tr. size	Model	Inference time/sample	mAP	mAR	mAA	mAF1
Teacher	Clean labels	19,997	ConvNext	5.04 ms	0.76554	0.74104	0.99174	0.75198
			RegnetX	5.08 ms	0.75315	0.72520	0.98984	0.73597
			VIT	3.6 ms	0.77396	0.75509	0.99244	0.76366
			Swin V2	5.52 ms	0.76759	0.75541	**0.99294**	0.75917
			Ensemble	20.04 ms	**0.80506**	**0.78418**	0.99174	**0.79269**
**Student**	Pseudo labels	221,629	ConvNext	5.04 ms	0.79554	0.76104	0.99174	0.76198
			RegnetX	5.08 ms	0.77315	0.74520	0.98984	0.74597
			VIT	3.6 ms	0.79396	0.77509	0.99244	0.77366
			Swin V2	5.52 ms	0.79759	0.78541	**0.99294**	0.76917
			Ensemble	20.04 ms	**0.83457**	**0.82899**	0.99174	**0.80880**

Significant values are given in bold.

**Table 4:** Comparison of strategies to enhance model performance in self-training.

Experiment	Labelling strategy	Tr. size	Model	Inference time/sample	mAP	mAR	mAA	mAF1
Label smoothing	Pseudo labels	221,629	ConvNext	5.04 ms	0.79554	0.76104	0.99174	0.76198
			RegnetX	5.08 ms	0.77315	0.74520	0.98984	0.74597
			VIT	3.6 ms	0.79396	0.77509	0.99244	0.77366
			Swin V2	5.52 ms	**0.79759**	**0.78541**	**0.99294**	0.76917
			Ensemble	20.4 ms	0.78920	0.77857	0.98565	**0.78741**
Weighted data loaders	Pseudo labels	221,629	ConvNext	5.04 ms	0.84578	0.83104	0.99568	0.79835
			RegnetX	5.08 ms	0.82290	0.81520	0.99112	0.76922
			VIT	3.6 ms	0.83645	0.82509	**0.99823**	0.78124
			Swin V2	5.52 ms	0.84595	0.83541	0.99676	0.79978
			Ensemble	20.04 ms	**0.86569**	**0.84457**	0.99725	**0.85880**

The reported results are based on the performance of the student model trained using pseudo labels.

Significant values are given in bold.

#### Augmenting self-training using weighted data loaders

As discussed in the dataset description section, our dataset not only suffers from noisy labels but also faces a significant class imbalance, posing the risk of biasing model development towards dominant classes while neglecting minority labels. To address this challenge, we implemented a Weighted Data Loader strategy (WDLs) that assigns weights to input samples based on the distribution of unique class label combinations in the dataset. This approach prioritises minority classes during batch selection by the data loaders in the training process. Our strategy computes the reciprocal of the logarithm of the frequency of class label combinations to inform WDLs ranking, instructing the batch processor to retrieve more samples from minority classes more frequently. It’s important to note that the WDLs strategy is not employed for training models from scratch but rather for fine-tuning existing models to specifically learn the minority classes over a few additional epochs. We integrated this strategy into both individual models and ensemble models within our self-training framework. Additionally, we froze the models’ bodies except the head to avoid catastrophic forgetting. The results of our analysis, summarised in Table 4, demonstrate the performance improvement achieved by this method. On average, the ensemble model showed a 5% enhancement in F1-score, a 3% increase in precision, and a 2% increase in recall when incorporating WDLs. Similarly, all individual models experienced performance improvements of 4-5% across various metrics. These findings underscore the effectiveness of incorporating weights during student model training within our self-training framework, leading to enhanced model robustness and performance. It is worth noting that the WDLs method is exclusively applied during the training of the student models.

### Statistical analysis

To assess the statistical significance of the differences in performance across the various methods in our proposed solution, we performed a one-way ANOVA test for each evaluation metric namely, mAP, mAR, mAA, and mAF1. The ANOVA results are presented in Table 5, which show that for mAP, mAR, and mAA, the ANOVA indicates statistically significant differences between the methods (p-value < 0.05). This implies that the different methods perform differently on these metrics, suggesting that the choice of method has a meaningful impact on performance. In contrast, the mAF1 metric showed a p-value slightly above the typical 0.05 threshold, this indicates that the observed differences between the methods for this metric are not statistically significant. To further investigate these differences, we applied the Kruskal-Wallis test (Table 5). The Kruskal-Wallis results confirmed the ANOVA findings for mAP and mAR, with p-values of 0.0068 and 0.0090, respectively, this highlights the presence of significant statistical differences. However, in the case of mAA, the Kruskal-Wallis test yielded a p-value of 0.1286, which suggests that the differences detected by ANOVA might not be statistically significant at the conventional level. Similarly, for mAF1, the Kruskal-Wallis test also indicated marginal significance with a p-value of 0.0868. Moreover, to identify which specific pairs of methods differed significantly, we performed Tukey’s Honestly Significant Difference (HSD) test. The results for this analysis are presented in Table 6, which demonstrates that significant pairwise differences can be observed between Label Smoothing and WDLs for mAP, mAR, and mAA, with p-values indicating clear differences. In contrast, the pairs of methods such as Label Smoothing and Student and Teacher and Student, did not show statistically significant differences. In summary, the statistical tests reveal that different methods exhibit significant variations in performance for most metrics, providing insights into their relative effectiveness.

**Table 5:** ANOVA and Kruskal-Wallis results for different evaluation metrics across the compared methods, i.e., models trained using the following settings: teacher, student, label smoothing, and weighted data loaders.

Metric	ANOVA		Kruskal-Wallis	
	F-statistic	p-value	Chi-square	p-value
mAP	**14.87**	**6.90** $\times$ ${\textbf {10}}^{{\textbf {-5}}}$	12.18	0.0068
mAR	**12.18**	**0.000211**	**11.58**	0.0090
mAA	5.53	0.00844	5.67	0.1286
mAF1	2.71	0.07996	6.57	0.0868

The bold values in the table indicate statistically significant results (p-value < *0.05)*.

**Table 6:** Tukey’s honestly significant difference (HSD) results for pairwise comparisons of methods across different metrics.

Metric	Group 1	Group 2	Mean diff.	p-value	Lower CI	Upper CI
mAP	Label smoothing	Student	0.0091	0.8421	− 0.0224	0.0406
	Label smoothing	Teacher	− 0.0168	0.4444	− 0.0483	0.0147
	Label smoothing	WDLs	**0.0535**	**0.0009**	**0.0220**	**0.0850**
	Student	Teacher	− 0.0259	0.1273	− 0.0574	0.0056
	Student	WDLs	**0.0444**	**0.0048**	**0.0129**	**0.0759**
	Teacher	WDLs	**0.0703**	**0.0000**	**0.0388**	**0.1018**
mAR	Label smoothing	Student	0.0101	0.8795	− 0.0289	0.0491
	Label smoothing	Teacher	− 0.0169	0.6126	− 0.0559	0.0221
	Label smoothing	WDLs	**0.0612**	**0.0019**	**0.0222**	**0.1002**
	Student	Teacher	− 0.0270	0.2367	− 0.0660	0.0120
	Student	WDLs	**0.0511**	**0.0085**	**0.0121**	**0.0901**
	Teacher	WDLs	**0.0781**	**0.0002**	**0.0391**	**0.1171**
mAA	Label smoothing	Student	0.0012	0.8166	− 0.0028	0.0052
	Label smoothing	Teacher	0.0012	0.8166	− 0.0028	0.0052
	Label smoothing	WDLs	**0.0053**	**0.0076**	**0.0013**	**0.0093**
	Student	Teacher	0.0000	1.0000	− 0.0040	0.0040
	Student	WDLs	**0.0041**	**0.0438**	**0.0001**	**0.0080**
	Teacher	WDLs	**0.0041**	**0.0438**	**0.0001**	**0.0080**
mAF1	Label smoothing	Student	0.0043	0.9923	− 0.0399	0.0485
	Label smoothing	Teacher	− 0.0069	0.9688	− 0.0511	0.0373
	Label smoothing	WDLs	0.0338	0.1680	− 0.0104	0.0780
	Student	Teacher	− 0.0112	0.8851	− 0.0554	0.0330
	Student	WDLs	0.0296	0.2614	− 0.0146	0.0738
	Teacher	WDLs	0.0408	0.0758	− 0.0034	0.0850

The bold values in the table indicate statistically significant results (p-value < 0.05).

## Conclusions

In this paper, we tackled the challenge of training robust models for surgical tool classification in endoscopic surgical videos, particularly when dealing with imbalanced datasets containing significant label noise. Our approach involved developing a systematic methodology to train robust machine learning (ML) models for diverse computer vision tasks. Our proposed systematic methodology initiates with training baseline models to identify the hardest or noisiest examples in the dataset for manual labelling. Subsequently, we introduced an efficient methodology for creating a clean dataset from a minimal sample size through active learning (AL), enabling us to train teacher models for pseudo-labelling the dataset. This pseudo-labelled dataset is then utilised to train student models for surgical tool classification, working in conjunction with the teacher models in a self-supervised learning manner. Throughout this methodology, we employed ensembling techniques for training the baseline, AL, teacher, and student models. The teacher model was trained using a manually labelled subset of cleaned data, while the student model leveraged a pseudo-labelled dataset generated by the teacher model, considering unclean (noisy) data as unlabelled examples. This ensembling approach proved effective in training robust models despite the presence of noisy labels in the dataset. To address the challenge of high-class imbalance, we introduced class weights during the self-supervised learning training process, resulting in a notable performance improvement of approximately 3–5% across different performance metrics. Looking ahead, our future work involves the development of an unsupervised learning-based methodology to address the challenge of training robust object detection and localisation models from such noisy data. We aim to explore explainable AI methods to generate heatmaps when the model classifies a tool, facilitating the identification and localisation of tools in the surgical scene without the need for bounding box annotated tools datasets, which are challenging to generate for computer vision applications in this domain. By delving into unsupervised learning techniques, we anticipate further enhancing the robustness and performance of our models in the face of diverse data quality issues.

## Data Availability

The data used for experiments in this article is acquired from the SurgToolLoc challenge (https://surgtoolloc23.grand-challenge.org/). Data annotated in this paper will be made available from the corresponding author upon reasonable request.
